# Disseminated Chickenpox Following Live Varicella Vaccination in a Crohn's Disease Patient on Combination Immunosuppression

**DOI:** 10.1155/crgm/6088333

**Published:** 2025-01-20

**Authors:** Quintin Solano, Sarah Uttal, Peter D. R. Higgins, Jeffrey A. Berinstein

**Affiliations:** ^1^Department of Internal Medicine, Michigan Medicine, Ann Arbor, Michigan, USA; ^2^Department of Gastroenterology and Hepatology, Michigan Medicine, Ann Arbor, Michigan, USA

## Abstract

Novel therapeutics used in the treatment of inflammatory bowel disease pose an increased risk of viral reactivation in patients. We present a case of a patient with refractory Crohn's disease (CD) who developed primary varicella (chickenpox) of a vaccine-viral strain after receiving combination immunosuppression with high-dose corticosteroids, tumor necrosis factor inhibitor (TNFi), and a Janus kinase inhibitor (JAKi) in the hospital. While this patient recovered and did not experience long term adverse effects, her case provides an opportunity for improvement. To improve safety, healthcare facilities should develop protocols that use electronic medical records enhanced with clinical decision support systems to identify and protect immunocompromised patients from inappropriate live vaccinations.

## 1. Background

Administering live vaccines in clinically significant immunocompromised patients is contraindicated due to the potential risk of virus reactivation [1, 2]. However, recent evidence challenges this perspective, highlighting the safe application of live varicella-zoster vaccine (VZV) in individuals receiving tumor necrosis factor inhibitor (TNFi) therapy [3]. The clinical landscape is evolving with the adoption of combination immunomodulatory strategies to manage refractory inflammatory bowel disease (IBD) patients [4–7]. Notably, recently approved novel small molecule classes, such as Janus kinase inhibitors (JAKi), have been associated with a significantly higher risk for VZ reactivation after prior varicella infection or vaccination [8–10]. Little is known about the risk of primary varicella infection after live vaccination in patients on combination immunosuppression including a JAKi. We present a case of a patient with refractory Crohn's disease (CD) who developed primary varicella (chickenpox) after receiving combination immunosuppression with high-dose corticosteroids, TNFi (infliximab), and a JAKi (upadacitinib).

## 2. Case Report

**Figure 1:**
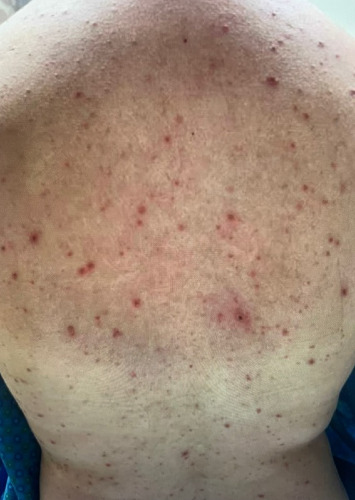
Patient's skin with crusting erosions at various stages of healing over the back.

**Figure 2:**
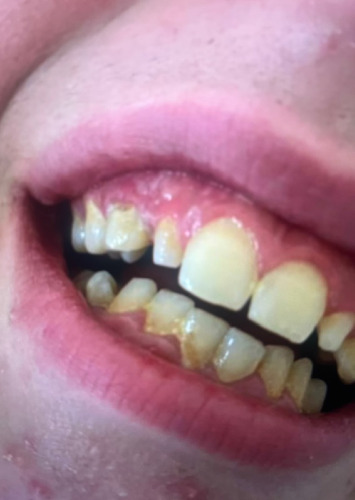
Vesicular lesion along the patient's gingiva.

**Figure 3:**
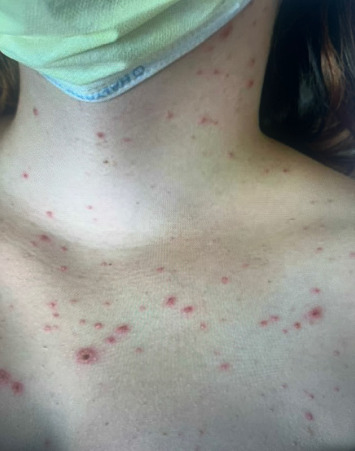
Patient's skin with crusting erosions at various stages of over the neck and chest.

A 24-year-old woman with a history of ileocolonic CD and previous total abdominal colectomy with ileorectal anastomosis for fulminant medical-refractory disease presented with hematochezia. Her CD had been previously maintained on methotrexate and infliximab; however, due to a lapse in insurance coverage, she was off therapy for 3 years. She was initiated on intravenous methylprednisolone 30 mg twice daily. Flexible enteroscopy on admission demonstrated severe inflammation characterized by diffuse ulceration of the remaining 20 cm of rectum to the anastomosis which was evaluated and intact. Biopsies demonstrated chronic inflammation without evidence of cytomegalovirus (CMV). In preparation for inpatient rescue therapy, she received the pneumococcal and hepatitis B vaccines in addition to inadvertently receiving the live varicella vaccine (Varivax). This vaccine was ordered by the primary team and approved by pharmacy at that time. After the administration of Varivax, the infectious disease team was consulted and recommended against prophylaxis prior to the initiation of infliximab due to clinical trial data that found no cases of varicella (chickenpox) or herpes zoster (HZ) (shingles) after administration of the live attenuated zoster vaccine (ZVL) in patients receiving TNFi therapy. Two days later, she received her first inpatient infliximab infusion followed by a second infusion 5 days later given ongoing severe symptoms. She remained symptomatic and repeat flexible enteroscopy demonstrated unchanged severe inflammation to the anastomosis. At this point, a permanent ileostomy was recommended; however, the patient strongly opposed. High-intensity off-label upadacitinib 30 mg twice daily was initiated 12 days after the administration of the live-varicella vaccine. Within 2 days, the patient experienced improvement in symptoms including complete resolution of her hematochezia. Despite improvement in CD symptoms, she reported numbness, tingling, bilateral shooting neuralgia in her lower extremities as well as back pain. The infectious disease team was reconsulted to assess for possible infectious explanations including varicella infection. Given her lack of infectious symptoms, rash, or clear explanation, her symptoms were attributed to deconditioning related to her prolonged hospital stay. The patient was discharged on a prednisone taper and standard dose upadacitinib 45 mg daily. Two weeks later, the patient presented with an extensive pruritic vesicular rash with crusting and erosions which involved her skin and mucous membranes (Figures 1, 2, and 3). Vesicle swabs were positive for VZV. She was diagnosed with primary varicella infection from vaccine viral strain and treated with acyclovir. After 4 months follow-up, the patient was doing well with complete resolution of her VZV symptoms, including her rash. She was vaccinated for varicella as a child.

## 3. Discussion

We present a rare case of disseminated varicella from the attenuated vaccine virus in a CD patient receiving combination immunosuppression with high-dose corticosteroids, TNFi, and a JAKi. This case provides an opportunity for several important clinical lessons for managing IBD in the modern era.

Varicella vaccine virus infection leading to primary varicella is extremely rare, whereas vaccine-associated shingles is more common. Rarely, patients can develop disseminated varicella, meningitis, or encephalitis from a vaccine virus and have been reported primarily in immunocompromised hosts. More commonly, patients can develop breakthrough varicella (chickenpox from a wildtype strain) or HZ infection with the vaccine strain of the virus [11, 12].

There is a paucity of data regarding the risk of vaccine related complications in the setting of combination immunosuppressive agents for refractory disease. It is known that autoimmune disease is an independent risk factors for opportunistic infections including primary varicella and secondary HZ reactivation; however, this risk increases significantly with the use of immunosuppressive agents. For example, the relative risk of HZ is 1.28–1.81 higher on TNFi therapy, 1.5–1.72 times on corticosteroid therapy, and 2.16 times higher on tofacitinib (a JAKi) [8]. Importantly, the risk for HZ increases as the tofacitinib dose increases. Our patient received three of these agents during her hospitalization and was on high-intensity off-label dosing of upadacitinib, which likely all contributed to her vaccine associated infection. This differential risk among agents is likely directly related to their mechanisms of action. In the immune response to VZV, signaling occurs via the JAK-STAT pathway [8, 13, 14]. Therefore, therapies targeting JAK complexes likely disrupt our immune response to primary VZV infection and may potentially increase the risk of VZV reactivation. While the infectious disease team cleared us for the use of a TNFi, they could not account for the potential need for additional immunosuppression agents. In the modern era of IBD treatment, it is becoming more common.

Generally, it is recommended that patients wait 2–6 weeks after live VZV vaccination before starting immunosuppression including a JAKi [15]. However, it can be difficult to predict which agents will be necessary to control severe IBD during hospitalization. In our case, the TNFi did not provide sufficient response, and the patient elected to move forward with JAKi therapy over the offered surgical options. A solution to avoid the inadvertent administration of a live vaccine in this patient population is to implement a hard stop on orders for vaccines in patients with a diagnosis of IBD and require a specialist's approval prior to approving the order (i.e., pharmacy and infectious disease).

This case stresses the importance of accurate knowledge about contraindications to live vaccines for healthcare providers, as well as the establishment of safety systems to prevent medical errors. With the increasing cases of IBD and new immunosuppressive drugs coming into use, there is a crucial need for improved education and safety checks in healthcare practices. To improve safety, healthcare facilities should develop protocols that use electronic medical records enhanced with clinical decision support systems to identify and protect immunocompromised patients from inappropriate live vaccinations. Additionally, more in-depth training for non-IBD specialists is necessary to keep them informed about new treatments for IBD and the associated risks concerning live vaccinations. Implementing these technological and educational measures into the daily operations of healthcare can significantly decrease the occurrence of adverse events and bolster patient safety and confidence in healthcare systems.

## Data Availability

The data that support the findings of this study are available from the corresponding author upon reasonable request.
